# COVID-19 among migrants, refugees, and IDPs: a synthesis of the global empirical literature

**DOI:** 10.1093/eurpub/ckac131.517

**Published:** 2022-10-25

**Authors:** M Hintermeier, N Gottlieb, J Oppenberg, A Mohsenpour, S Flores, S Rohleder, S Pernitez-Agan, J Lopez, K Wickramage, K Bozorgmehr

**Affiliations:** 1 Department of General Practice and HSR, University Hospital Heidelberg, Heidelberg, Germany; 2 Departmentof Population Medicine and Health Research, Bielefeld University, Bielefeld, Germany; 3 Department of Public Health and Caring Sciences, Uppsala University, Uppsala, Sweden; 4 UN Migration Agency, International Organization for Migration, Manila, Philippines

## Abstract

**Background:**

The COVID-19 syndemic reveals social and health inequalities, putting marginalized groups such as migrants at greater risk. Yet health systems fail to routinely monitor the health of migrants, refugees, and internally displaced persons. Our systematic review provides an up-to-date synthesis of the empirical evidence on COVID-19 infection risk, transmission, outcome of disease and risk of severe course of disease among migrant populations. It further aims to compile extant evidence on COVID-19 vaccination coverage among these groups, and on the effects of pandemic control measures on their health.

**Methods:**

Following PRISMA guidelines, we registered a review protocol, searched 14 scientific databases and 4 pre-print servers using the WHO database of global literature on COVID-19, and hand-searched relevant websites for grey literature. The search period covers the time from 12/2019 to 11/30/2021. Articles in English, German and Spanish and all study designs were included.

**Results:**

A total of 6966 references were identified for title and abstract screening. 518 records were screened in full-text, out of which 204 articles were included so far (conflict solving at full-text stage and data extraction are ongoing). Our review presents a broad landscape of different study designs, migrant populations and COVID-19 outcomes. Based on previous work, we expect to find a higher risk of infection in migrants and their disproportionate share among COVID-19 cases, and consolidate the (mental) health impacts of pandemic control measures. Our preliminary findings indicate a vast knowledge gap on vaccination coverage among migrant groups.

**Conclusions:**

Two years into the syndemic, this review summarizes the global empirical evidence on the impact of the COVID-19 syndemic on migrant populations. With health systems often lacking related data, the review provides an important evidence base for the consideration of migrants in future pandemic preparedness policies.

**Key messages:**

• The systematic review provides an up-to-date synthesis of the empirical evidence on COVID-19 among diverse migrant populations globally.

• Knowledge on vaccination coverage in migrants remains patchy.

